# Supplementary material for: Genetic and epigenetic intratumor heterogeneity impacts prognosis of lung adenocarcinoma
Source: Nat Commun. 2020 May 18;11:2459. doi: 10.1038/s41467-020-16295-5 (PMC7235245; doi:10.1038/s41467-020-16295-5)
Supplement: Supplementary file 1 — Supplementary Information [file 41467_2020_16295_MOESM1_ESM.pdf]

Supplementary Information

Genetic and Epigenetic Intratumor Heterogeneity Impacts Prognosis of Lung Adenocarcinoma

Hua et al.

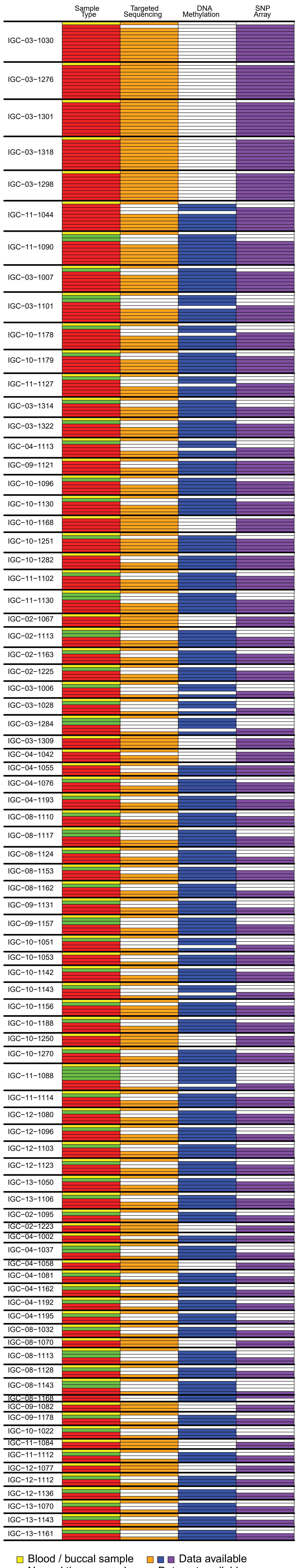

Supplementary Figure 1. Description of the experiments (targeted sequencing, DNA methylation and SNP array) conducted on each sample. The thick black lines separate the tumors. Each row within the thick lines correspond to a sample. Multiple rows between thick lines indicate multiple samples from the same tumor. The different colors indicate different sample types: blood/buccal (yellow), normal lung tissue (green) and tumor lung tissue (red).

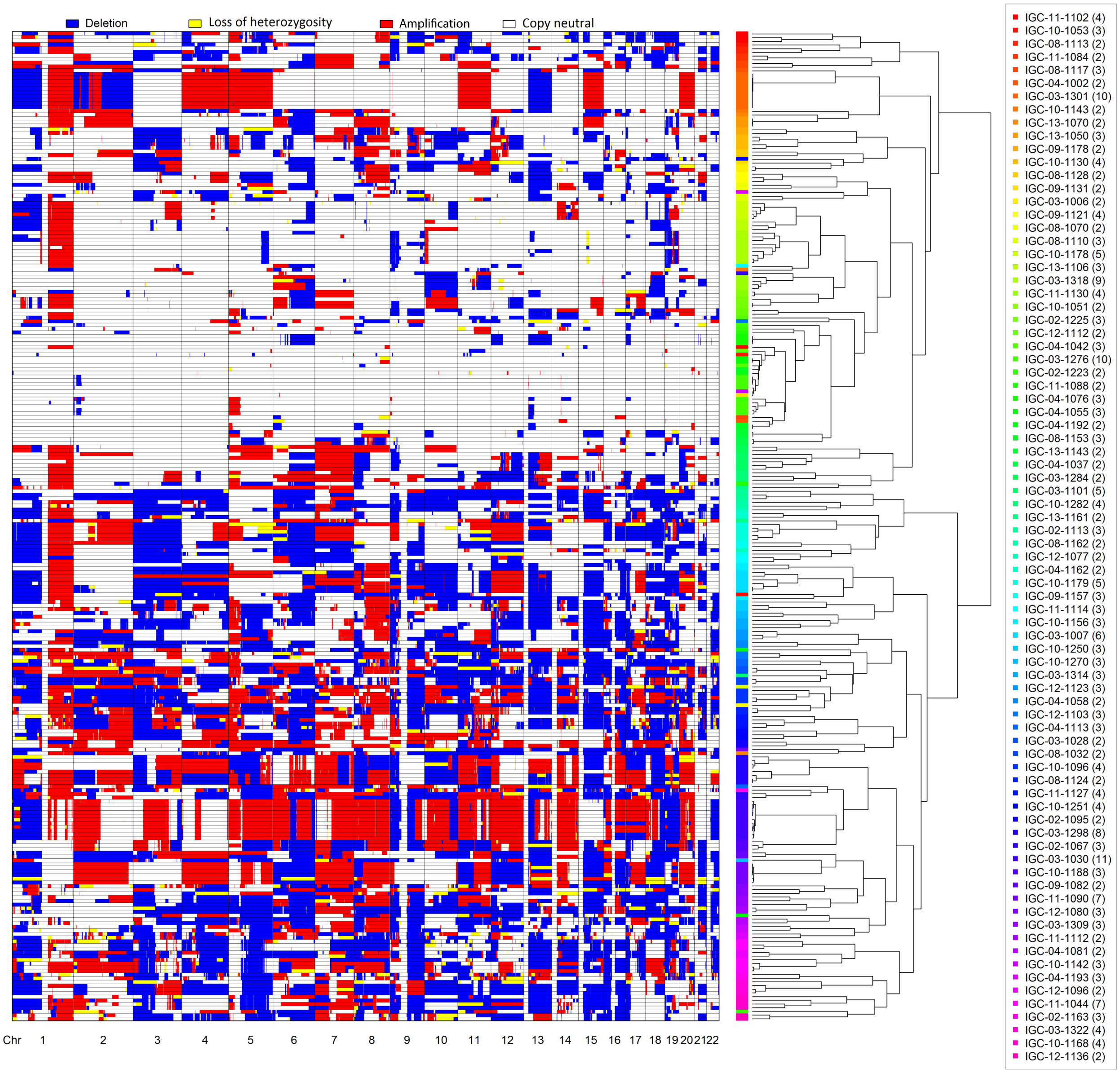

Supplementary Figure 2. Unsupervised clustering of somatic copy number alteration (SCNA) profiles of 80 tumors using multi-region sampling. Heatmap colors represent different SCNA event types: blue indicates deletion, yellow indicates loss of heterozygosity, red indicates amplification, and white indicates copy number neutral. Different tumors are indicated by different colors in the row sidebar.

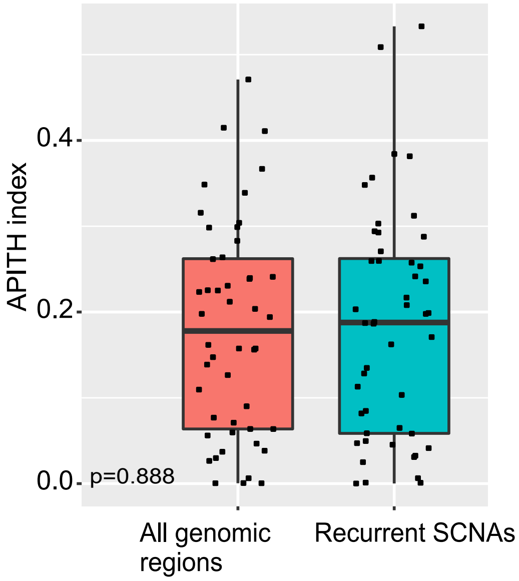

Supplementary Figure 3. Comparison of intratumor heterogeneity (ITH) of SCNAs across the whole genome (left) and SCNAs in recurrently altered regions (right) based on TCGA studies[2,13] in lung tumor samples. The center line indicates median APITH index. The box length indicates the interquartile range. The whiskers extend to the largest and smallest APITH. The p-value is based on two-sided Student's t-test. For both groups, n=48 tumors with at least 3 samples.

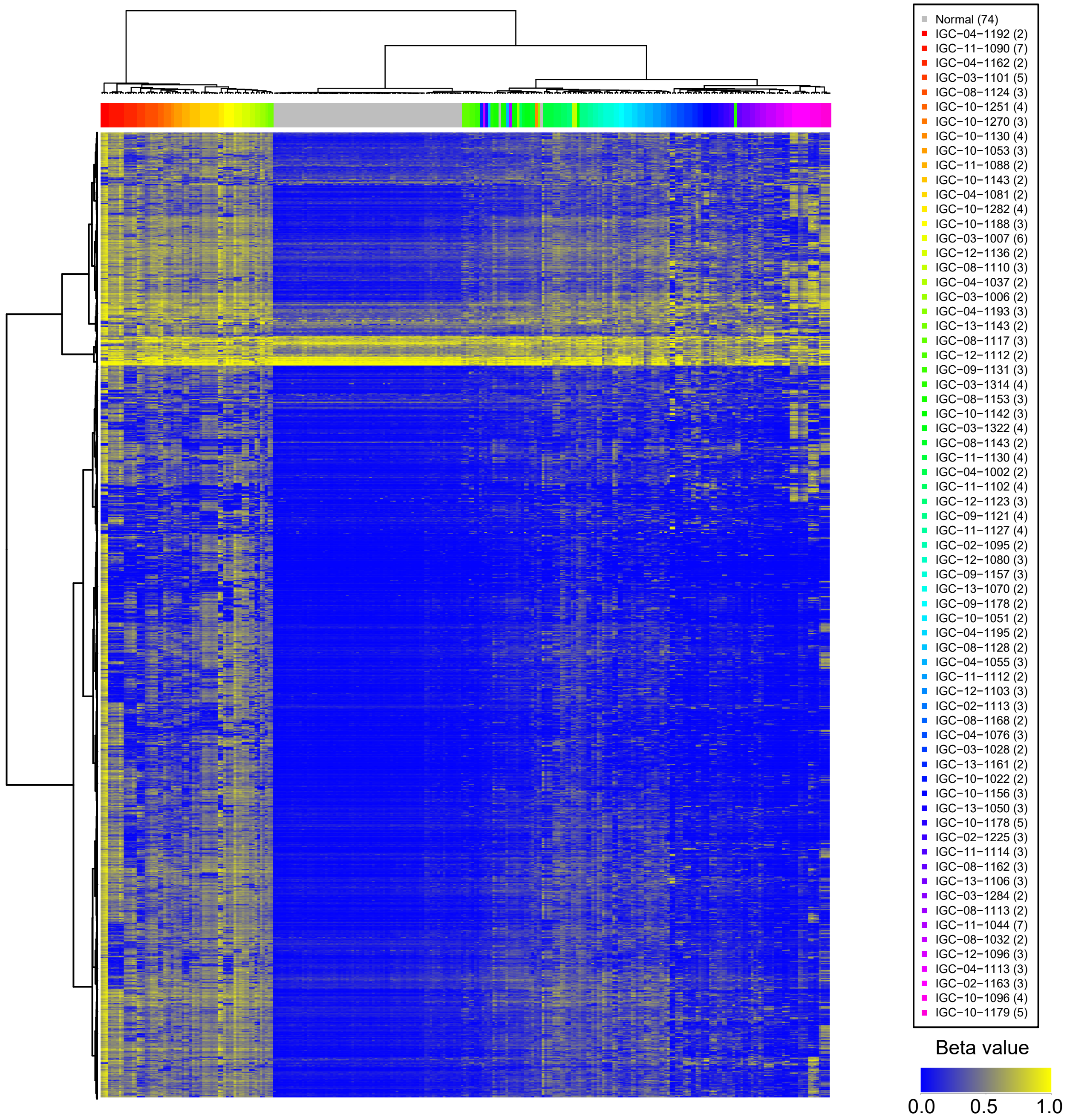

Supplementary Figure 4. Intratumoral heterogeneity of DNA methylation profiles. Unsupervised hierarchical clustering of 5000 most variable probes in CpG islands in the promoter regions from 68 subjects. Different tumors are indicated by different colors in the column sidebar, with normal samples colored in gray. The numbers in parenthesis are the number of normal tissue samples for the 'normal' group, or the number of tumor samples in each patient. The beta values represent estimates of methylation levels, with 0 being unmethylated and 1 fully methylated.

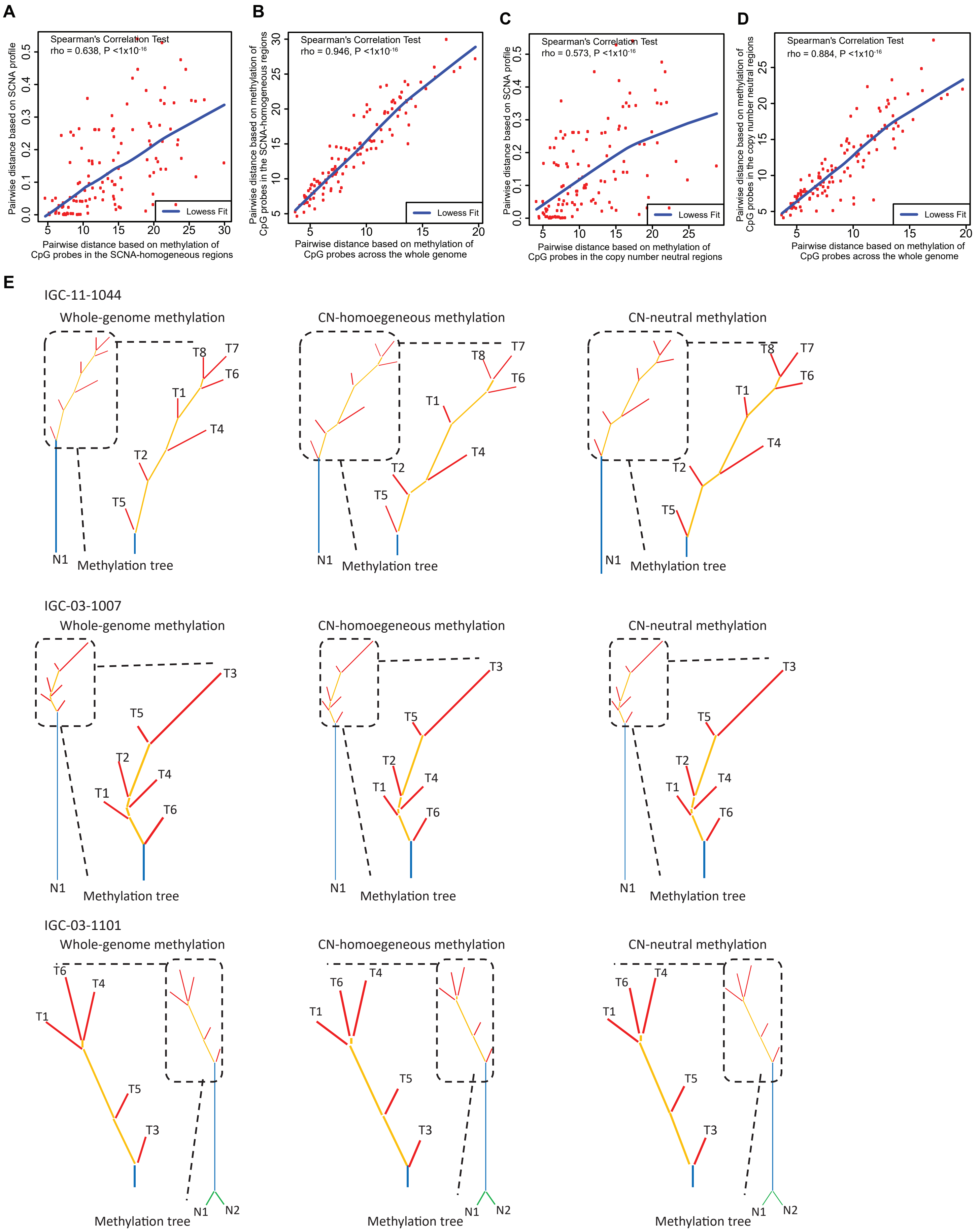
A
Spearman's Correlation Test
rho = 0.638, P <1x10-16
Pairwise distance based on SCNA profile
Pairwise distance based on methylation of CpG probes in the SCNA-homogeneous regions
Lowess Fit
B
Spearman's Correlation Test
rho = 0.946, P <1x10-16
Pairwise distance based on methylation of CpG probes in the SCNA-homogeneous regions
Pairwise distance based on methylation of CpG probes across the whole genome
Lowess Fit
C
Spearman's Correlation Test
rho = 0.573, P <1x10-16
Pairwise distance based on SCNA profile
Pairwise distance based on methylation of CpG probes in the copy number neutral regions
Lowess Fit
D
Spearman's Correlation Test
rho = 0.884, P <1x10-16
Pairwise distance based on methylation of CpG probes in the copy number neutral regions
Pairwise distance based on methylation of CpG probes across the whole genome
Lowess Fit
E
IGC-11-1044
Whole-genome methylation
CN-homoegeneous methylation
CN-neutral methylation
T1 T2 T4 T5 T6 T7 T8
N1
Methylation tree
IGC-03-1007
Whole-genome methylation
CN-homoegeneous methylation
CN-neutral methylation
T1 T2 T3 T4 T5 T6
N1
Methylation tree
IGC-03-1101
Whole-genome methylation
CN-homoegeneous methylation
CN-neutral methylation
T1 T3 T4 T5 T6
N1 N2
Methylation tree

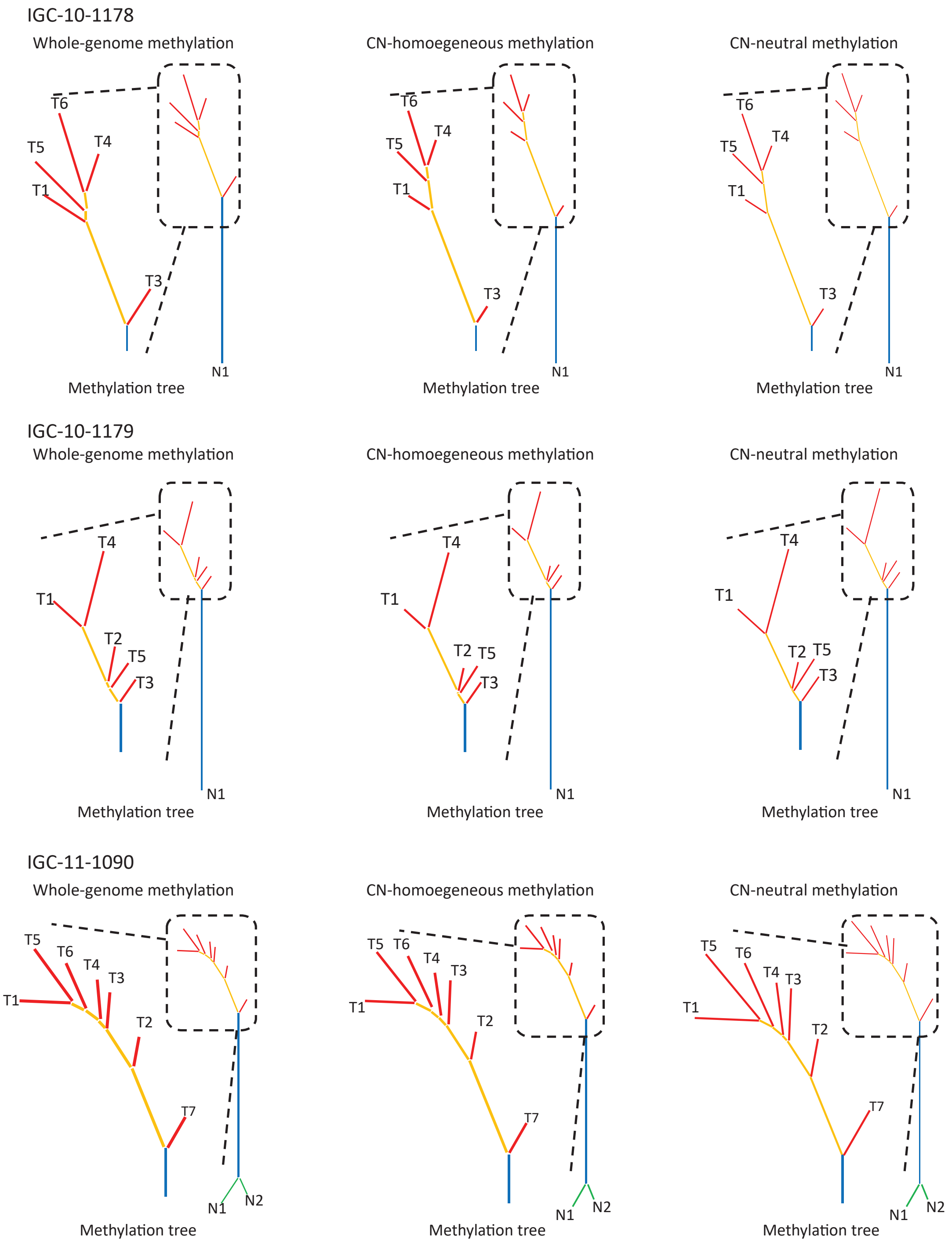

Supplementary Figure 5. Pairwise tumor sample distance and inferred evolutionary trajectories. Comparison of pairwise Euclidean distance and inferred evolutionary trajectories based on SCNAs and CpG probes in whole-genome, SCNA homogeneous regions and copy number neutral regions. (A-D, related to **Figure 6A.** n=212 sample pairs for each figure. The p-values are based on Spearman's correlation test.) For 14 tumors with at least 4 samples per tumor assayed for methylation, pairwise Euclidean distance of tumor samples from the same tumor based on (A) SCNAs and CpG probes in the SCNA homogeneous regions, (B) CpG probes in the SCNA homogeneous regions and whole-genome CpG probes, (C) SCNAs and CpG probes in the copy number neutral regions, (D) CpG probes in the copy number neutral regions and whole-genome CpG probes. (E, related to **Figure 6C**) For six tumors with at least five samples per tumor assayed for both SCNAs and DNA methylation, phylogenetic analysis based on CpG probes across the whole genome (left), CpG probes mapping to SCNA homogenous regions (middle) and CpG probes mapping to copy number neutral regions (right). Blue lines represent alterations shared by all tumor samples from the same subject. Yellow lines represent alterations shared by two or more tumor samples. Red lines represent alterations specific to one tumor sample. Green lines represent alterations specific to one normal sample.

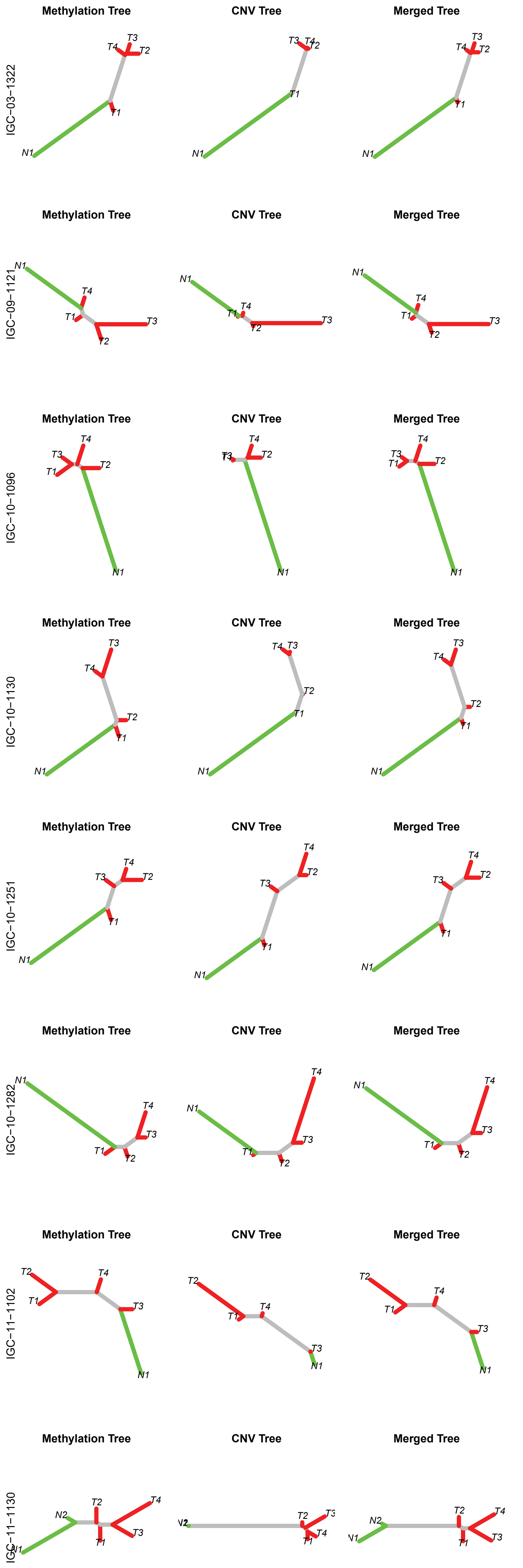

Supplementary Figure 6. Phylogenetic analysis for eight tumors with four samples assayed for both SCNAs and DNA methylation. Phylogenetic trees are based on methylation data (left), SCNA data (middle) and merged consensus tree (right). Green lines indicate alterations specific to one normal sample. Red lines indicate alterations specific to one tumor sample. Gray lines indicate alterations shared by multiple tumor samples.

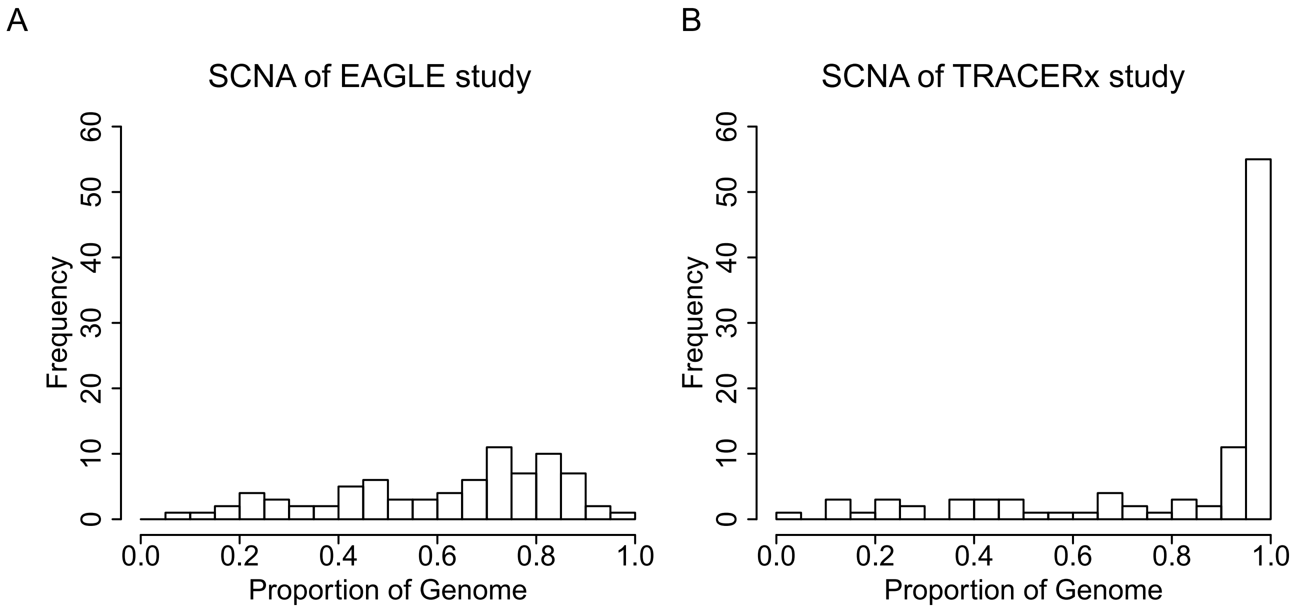

Supplementary Figure 7. Comparison of SCNA fraction. Distribution of the SCNA fraction of (A) 80 tumors in the EAGLE study and (B) 100 tumors in the TRACERx study. The SCNA fraction of a tumor was defined based on the proportion of the genome altered in at least one sample from each tumor (X axis).

Supplementary Note for "Genetic and Epigenetic Intratumor Heterogeneity of Lung Adenocarcinoma"

1. **Estimating the Variance of the APITH Estimator**

As we show in Figure 3B, the averaged pairwise ITH index (APITH) does not depend on the number of tumor samples for a patient. However, the variance of the APITH estimator depends on the number of tumor samples, which we call as measurement error. Different from the classic measurement error theory where the variance of the measurement error is constant across subjects, APITH has subject-specific variance. To achieve the optimal statistical power for testing the association between APITH and a clinical outcome, we incorporate the subject-specific variance as weight in a regression analysis. In this note, we methods to estimate such variances.

To proceed, we estimate the unconditional variance of APITH estimator, which is partitioned into two parts: the across-patient variance and the expected within patient variance. The weight for regression analyses is proportional to the reciprocal of the unconditional variance. In what follows, we estimate the unconditional variance of APITH.

Lemma 1. Given one subject with k tumor samples. Let d_{ij} be the pairwise distance between tumor samples i and j. Assume that $E[d_{ij}] = \mu$. We define $D = \sum_{1\le i<j\le k} d_{ij} / \binom{k}{2}$ as APITH. For the given subject, the variance of APITH for the given subject is derived as

$$Var(D|\mu) = \frac{2}{k(k-1)}Var(d_{ij}) + \frac{4(k-2)}{k(k-1)}Cov(d_{ij}, d_{jm}). \quad (1)$$

Here, (i, j, m) are distinct integers.

Proof. It is straightforward to show that $E(D) = \mu$. By definition, we have

$$Var(D|\mu) = ED^2 - (ED)^2 = \frac{E\left[\left(\sum_{1\le i<j\le k} d_{ij}\right)^2\right]}{\binom{k}{2}^2} - \mu^2. \quad (2)$$

Furthermore, we have

$$E\left(\sum_{1\le i<j\le k} d_{ij}\right)^2 = \binom{k}{2}E[d_{ij}^2] + k(k-1)(k-2)E[d_{ij}d_{im}] + \frac{1}{4}k(k-1)(k-2)(k-3)E[d_{ij}d_{ml}]. \quad (3)$$

Given the subject, $E[d_{ij}d_{kl}] = \mu^2$ because d_{ij} and d_{lk} are uncorrelated. Thus,

$$Var(D|\mu) = \frac{2}{k(k-1)} E\left[d_{ij}^2\right] + \frac{4(k-2)}{k(k-1)} E\left[d_{ij}d_{im}\right] + \frac{(k-2)(k-3)}{k(k-1)}\mu^2 - \mu^2$$
$$= \frac{2}{k(k-1)}\left(E\left[d_{ij}^2\right] - \mu^2\right) + \frac{4(k-2)}{k(k-1)}\left(E\left[d_{ij}d_{im}\right] - \mu^2\right)$$
$$= \frac{2}{k(k-1)} Var\left(d_{ij}\right) + \frac{4(k-2)}{k(k-1)} Cov\left(d_{ij}, d_{jm}\right).$$

This proves (1).

Comment. (1) is the conditional variance of the APITH estimator for a given subject, reflecting the measurement error of the ITH estimator due to small number of tumor samples. When number of tumor samples increases to infinity, the ITH for the subject will be measured without error. In what follows, we will derive the unconditional variance of the APITH estimator which will be used as the weight for regression analysis.

Unconditional variance of APITH

Suppose that we have a set of K patients. For subject s with k_s tumor samples, let $d_{s,ij}$ be the pairwise distance between two tumor samples i and j. We assume $Ed_{s,ij} = \mu_s$. Here, the expectation is calculated conditioning on subject s. For subject s, the APITH is calculated as

$$D_s = \frac{\sum_{1\le i<j\le k_s} d_{s,ij}}{\binom{k_s}{2}}. \tag{4}$$

By the variance decomposition formula, the unconditional variance is partitioned as

$$Var(D_s) = Var(E[D_s|\mu_s]) + E[Var(D_s|\mu_s)]. \tag{5}$$

Note that $E(D_s|\mu_s) = \mu_s$, *i.e.*,

$$Var(E[D_s|\mu_s]) = Var(\mu_s). \tag{6}$$

By lemma (1), we have

$$Var(D_s|\mu_s) = \frac{2}{k_s(k_s-1)} E\left[Var\left(d_{s,ij}|\mu_s\right)\right] + \frac{4(k_s-2)}{k_s(k_s-1)} E\left[Cov\left(d_{s,ij}, d_{s,jm}|\mu_s\right)\right]. \tag{7}$$

Now we propose estimators for the three items in (6) and (7).

First, we have

$$E\left[\left(d_{s,ij} - d_{s,ml}\right)^2|\mu_s\right] = E\left[\left(d_{s,ij} - \mu_s - d_{s,ml} - \mu_s\right)^2|\mu_s\right] = 2Var\left(d_{s,ij}|\mu_s\right);$$

thus,

$$E\left(Var\left(d_{s,ij}|\mu_s\right)\right) = \frac{1}{2} E\left(E\left[\left(d_{s,ij} - d_{s,ml}\right)^2|\mu_s\right]\right), \tag{8}$$

where the outer expectation is calculated with respect to μ_s and (i, j, m, l) are distinct integers. For a subject with at least four subjects, we estimated $E\left[\left(d_{s,ij} - d_{s,ml}\right)^2 | \mu_s\right]$ by

$$\frac{1}{\binom{k_s}{4}} \sum_{i,j,m,l} \left(d_{s,ij} - d_{s,ml}\right)^2.$$

Thus, (8) was estimated using patients (denoted as a set A) with at least four tumor samples

$$\frac{1}{|A|} = \sum_{s \in A} \frac{1}{\binom{k_s}{4}} \sum_{i,j,m,l} \left(d_{s,ij} - d_{s,ml}\right)^2. \tag{9}$$

Similarly, we can prove the following equality:

$$EE\left[\left(d_{s,ij} - d_{t,ij}\right)^2\right] = 2E\left(Var\left(d_{s,ij} | \mu_s\right)\right) + 2Var(\mu_s).$$

Thus,

$$Var(\mu_s) = \frac{1}{2} EE\left[\left(d_{s,ij} - d_{t,ij}\right)^2\right] - E\left(\mathrm{Var}\left(d_{s,ij} | \mu_s\right)\right). \tag{10}$$

The second term is estimated by (8) and (9). The first term is estimated using all pairs of tumor samples from all pairs of patients.

Finally, we can prove the following equality

$$EE\left[\left(d_{s,ij} - d_{s,ik}\right)^2\right] = 2E\left(Var\left(d_{s,ij} | \mu_s\right)\right) - 2E\left(Cov\left(d_{s,ij}, d_{s,im} | \mu_s\right)\right). \tag{11}$$

Here, (i, j, m) are distinct integers. Thus, we can first estimate $EE\left[\left(d_{s,ij} - d_{s,ik}\right)^2\right]$ using subjects with at least three tumor samples and then derive an estimate for $E\left(Cov\left(d_{s,ij}, d_{s,im} | \mu_s\right)\right)$ using an estimate of $E\left(Var\left(d_{s,ij} | \mu_s\right)\right)$ in (9).

2. Comparing TRACERx and this study using *APITH* for SCNAs

A previous TRACERx study [1], which analyzed SCNAs derived from whole exome sequencing data, reported a higher ITH for SCNA (median=43% in the 57 LUAD patients) compared to our study based on APITH (median=15.7%). This is likely because the ITH was calculated in different ways. The TRACERx study defined ITH as the fraction of SCNAs not shared by all samples in a tumor, which depends on the number of samples (Fig. 3B). In addition, the SCNA fraction was calculated using the SCNA-disrupted region as the denominator in the TRACERx study; in our study, the denominator was the whole genome.

To directly compare ITH values between the two studies, we downloaded the SCNA data from the TRACERx study and calculated *APITH* in the 57 LUAD patients. We found a much lower ITH in the TRACERx study (median=4.0%) compared to our study (median=15.7%) using APITH. This is likely caused by the fact that in the TRACERx study, over 50% of samples had whole genome disrupted by SCNAs. Since the estimation of the absolute copy number in amplified regions has low accuracy, we relied on only presence or absence of SCNAs for this comparison. Thus, having the whole genome disrupted like in most TRACERx samples would be estimated as having only one copy number event, resulting in extremely low ITH. (Supplementary Fig. 7).

1. Jamal-Hanjani, M. *et al.* Tracking the Evolution of Non-Small-Cell Lung Cancer. *N Engl J Med* **376**, 2109-2121 (2017).
