# Supplementary material for: Genetic and epigenetic intratumor heterogeneity impacts prognosis of lung adenocarcinoma
Source: Nat Commun. 2020 May 18;11:2459. doi: 10.1038/s41467-020-16295-5 (PMC7235245; doi:10.1038/s41467-020-16295-5)
Supplement: Supplementary file 3 — Description of Additional Supplementary Files [file 41467_2020_16295_MOESM3_ESM.pdf]

Description of Additional Supplementary Files

File Name: Supplementary Data 1
Description: Demographic and clinical characteristics of 84 lung adenocarcinoma patients.

File Name: Supplementary Data 2
Description: Summary of clonal and subclonal somatic mutations in 37 lung cancer driver genes.

File Name: Supplementary Data 3
Description: Summary of clonal and subclonal SCNAs of 33 cancer driver genes in recurrently altered genomic regions.

File Name: Supplementary Data 4
Description: Association of genomic and epigenomic ITH with clinical features.

File Name: Supplementary Data 5
Description: Cox regression analysis for overall survival and risk of metastasis stratified by ITH of SCNAs.

File Name: Supplementary Data 6
Description: Cox regression analysis for overall survival and risk of metastasis stratified by ITH of DNA methylation.
