# Supplementary material for: Genetic and epigenetic intratumor heterogeneity impacts prognosis of lung adenocarcinoma
Source: Nat Commun. 2020 May 18;11:2459. doi: 10.1038/s41467-020-16295-5 (PMC7235245; doi:10.1038/s41467-020-16295-5)
Supplement: Supplementary file 10 — Reporting Summary [file 41467_2020_16295_MOESM10_ESM.pdf]

nature research

Corresponding author(s): Maria Teresa Landi

Last updated by author(s): 3/31/2020

Reporting Summary

Nature Research wishes to improve the reproducibility of the work that we publish. This form provides structure for consistency and transparency in reporting. For further information on Nature Research policies, see Authors & Referees and the Editorial Policy Checklist .

Statistics

For all statistical analyses, confirm that the following items are present in the figure legend, table legend, main text, or Methods section.

n/a	Confirmed	
☐	☒	The exact sample size (*n*) for each experimental group/condition, given as a discrete number and unit of measurement
☐	☒	A statement on whether measurements were taken from distinct samples or whether the same sample was measured repeatedly
☐	☒	The statistical test(s) used AND whether they are one- or two-sided<br>*Only common tests should be described solely by name; describe more complex techniques in the Methods section.*
☐	☒	A description of all covariates tested
☐	☒	A description of any assumptions or corrections, such as tests of normality and adjustment for multiple comparisons
☐	☒	A full description of the statistical parameters including central tendency (e.g. means) or other basic estimates (e.g. regression coefficient) AND variation (e.g. standard deviation) or associated estimates of uncertainty (e.g. confidence intervals)
☐	☒	For null hypothesis testing, the test statistic (e.g. *F*, *t*, *r*) with confidence intervals, effect sizes, degrees of freedom and *P* value noted<br>*Give P values as exact values whenever suitable.*
☒	☐	For Bayesian analysis, information on the choice of priors and Markov chain Monte Carlo settings
☒	☐	For hierarchical and complex designs, identification of the appropriate level for tests and full reporting of outcomes
☐	☒	Estimates of effect sizes (e.g. Cohen's *d*, Pearson's *r*), indicating how they were calculated

Our web collection on statistics for biologists contains articles on many of the points above.

Software and code

Policy information about availability of computer code

Data collection	Deep targeted sequencing data: Ion Torrent Suite Software (Version 5.0.7). SNP arrays: Illumina GenomeStudio (Version 2011.1.0.24550).
Data analysis	R (version 3.6.0) was used for data analysis. R packages: minfi, ASCAT, dNdSCV,ape, InfiniumPurify, survey, ggplot2.<br>Custom algorithms have been deposited to https://github.com/xtmgah/EAGLE_LUAD.<br>Torrent variant caller (version 5.0.9).

For manuscripts utilizing custom algorithms or software that are central to the research but not yet described in published literature, software must be made available to editors/reviewers. We strongly encourage code deposition in a community repository (e.g. GitHub). See the Nature Research guidelines for submitting code & software for further information.

Data

Policy information about availability of data

All manuscripts must include a data availability statement. This statement should provide the following information, where applicable:
- Accession codes, unique identifiers, or web links for publicly available datasets
- A list of figures that have associated raw data
- A description of any restrictions on data availability

The targeted sequencing data have been deposited in SRA through dbGaP under the accession number phs001169.v2.p1. The SNP array and methylation array data have been deposited in dbGaP under the same accession number.

Field-specific reporting

Please select the one below that is the best fit for your research. If you are not sure, read the appropriate sections before making your selection.

Life sciences study design

All studies must disclose on these points even when the disclosure is negative.

Sample size	Tumor samples from 84 patients were confirmed as primary lung adenocarcinoma. 2 to 11 samples from each tumor were analyzed. Sample size was determined by the availability of the patients recruited.
Data exclusions	Samples with low purity were excluded from the study. No predetermined data exclusions based on age, sex or other subjects' characteristics was applied.
Replication	No replication was applied to individual samples. But multiple regions from the same tumor were profiled.
Randomization	We did not investigate the effect of different treatments, so it is not applicable to this study.
Blinding	We did not investigate the effect of different treatments, so it is not applicable to this study.

Reporting for specific materials, systems and methods

We require information from authors about some types of materials, experimental systems and methods used in many studies. Here, indicate whether each material, system or method listed is relevant to your study. If you are not sure if a list item applies to your research, read the appropriate section before selecting a response.

Materials & experimental systems

n/a	Involved in the study	
☒	☐	Antibodies
☒	☐	Eukaryotic cell lines
☒	☐	Palaeontology
☒	☐	Animals and other organisms
☐	☒	Human research participants
☒	☐	Clinical data

Methods

n/a	Involved in the study	
☒	☐	ChIP-seq
☒	☐	Flow cytometry
☒	☐	MRI-based neuroimaging

Human research participants

Policy information about studies involving human research participants

Population characteristics	Age, gender, smoking status, tumor stage, survival status and time to death, metastatic status and time to metastasis
Recruitment	The patients were recruited in the EAGLE study, a population-based case-control study conducted in Italy between 2002 and 2005.
Ethics oversight	The study protocol was approved by the Institutional Review Board of the US National Cancer Institute and the involved institutions in Italy. Informed consent was obtained for all subjects prior to study participation.

Note that full information on the approval of the study protocol must also be provided in the manuscript.
